# Bottom‐up Design of Bimetallic Cobalt–Molybdenum Carbides/Oxides for Overall Water Splitting

**DOI:** 10.1002/chem.201905265

**Published:** 2020-01-30

**Authors:** Rongji Liu, Montaha Anjass, Simon Greiner, Si Liu, Dandan Gao, Johannes Biskupek, Ute Kaiser, Guangjin Zhang, Carsten Streb

**Affiliations:** ^1^ Institute of Inorganic Chemistry I Ulm University Ulm 89081 Germany; ^2^ Center of Materials Science and Optoelectronics Engineering University of the Chinese Academy of Sciences Beijing 100049 P. R. China; ^3^ Institute of Process Engineering, Key Laboratory of Green Process and Engineering Chinese Academy of Sciences Beijing 100190 P. R. China; ^4^ Helmholtz-Institute Ulm Electrochemical Energy Conversion Ulm 89081 Germany; ^5^ Central Facility of Electron Microscopy for Materials Science, Ulm University Albert-Einstein-Allee 11 Ulm 89081 Germany

**Keywords:** composites, electrocatalysis, polyoxometalates, self-assembly, water splitting

## Abstract

Earth‐abundant transition‐metal‐based catalysts for electrochemical water splitting are critical for sustainable energy schemes. In this work, we use a rational design method for the synthesis of ultrasmall and highly dispersed bimetallic CoMo carbide/oxide particles deposited on graphene oxide. Thermal conversion of the molecular precursors [H_3_PMo_12_O_40_], Co(OAc)_2_ **⋅**4 H_2_O and melamine in the presence of graphene oxide gives the mixed carbide/oxide (Co_6_Mo_6_C_2_/Co_2_Mo_3_O_8_) nanoparticle composite deposited on highly dispersed, N,P‐doped carbon. The resulting composite shows outstanding electrocatalytic water‐splitting activity for both the oxygen evolution and hydrogen evolution reaction, and superior performance to reference samples including commercial 20 % Pt/C & IrO_2_. Electrochemical and other materials analyses indicate that Co_6_Mo_6_C_2_ is the main active phase in the composite, and the N,P‐doping of the carbon matrix increases the catalytic activity. The facile design could in principle be extended to multiple bimetallic catalyst classes by tuning of the molecular metal oxide precursor.

## Graphical abstract

**A rational bottom‐up design** method gives access to bimetallic metal carbide/oxide nanoparticles deposited on N,P‐doped carbon matrices. The resulting composites show outstanding electrochemical reactivity for oxygen and hydrogen evolution and allow the assembly of an overall water‐splitting device (see figure).

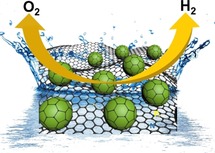

## Introduction

Electrochemical water splitting involving the hydrogen evolution reaction (HER) at the cathode and oxygen evolution reaction (OER) at the anode is a promising energy conversion technology that can convert intermittent electricity into storable chemical energy, that is, H_2_. Earlier, noble metals, such as Pt have been utilized as the most efficient electrocatalysts for HER and noble metal oxides, for example, IrO_2_ or RuO_2_ have been used as the electrocatalysts for OER. However, prohibitive costs and low availability of these materials hinder their large‐scale industrial deployment. Therefore, the development of earth‐abundant transition‐metal‐based materials for these challenging reactions is a critical task for materials chemistry.^1^, ^2^, ^3^ Over the last decade, transition‐metal oxides,^4^ carbides,^5^, ^6^ nitrides,^7^ sulfides,^8^ and phosphides^9^, ^10^ have been reported as promising catalysts for water electrolysis. However, many of these materials are hampered by low reactivity or low stability under the conditions employed technologically in alkaline water electrolysis. In addition, few of these materials can be used as bifunctional catalysts, that is, to perform both OER and HER. To address these issues, new, bimetallic oxides,^11^, ^12^, ^13^, ^14^ hydroxides,^15^, ^16^, ^17^ carbides,^18^, ^19^ nitrides,^20^ and phosphides^21^, ^22^ have been developed to improve reactivity and stability for full water splitting.^23^

Amongst these materials, the late transition‐metal carbides (LTMC, for example, Mo_2_C, MoC, W_2_C, WC etc.) are considered benchmark materials and have shown promising HER activity, especially in acid, due to a similar electronic structure to Pt‐group metals.^5^, ^24^, ^25^, ^26^, ^27^, ^28^, ^29^, ^30^ However, it is still challenging to use those materials as catalysts in the alkaline water electrolyzer due to their low activity for OER under these conditions. Current research is trying to overcome this issue either by doping a secondary early transition metal (ETM, for example, Co, Ni etc.) into the LTMC^31^, ^32^, ^33^, ^34^, ^35^ or by direct synthesis of bimetallic transition metal carbides (BTMC) that contain secondary ETMs, thus greatly enhancing the water‐splitting activity.^19^, ^36^ The straightforward method for preparing the BTMC is the one‐pot calcination of metal precursors (mixtures of ETM and LTM based precursors) and carbon source. However, as most of these syntheses require high‐temperature pyrolysis, the preparation of pure BTMC phases is difficult to achieve, and further, agglomeration of the particles formed often results in poor catalytic activity. Therefore, it is still a challenge to synthesize pure phase ultrasmall bimetallic carbides with high dispersion as high‐performance water‐splitting electrocatalysts.

To further increase the electrical conductivity of the materials, and thus to increase the reactivity of the materials for electrocatalysis, the deposition of the carbides on high surface‐area conductive carbons (mesoporous carbon, carbon nanotubes (CNTs) and graphene etc.) is a key concept.^1^, ^34^, ^37^

Herein, we propose a rational design method for the synthesis of ultrasmall N,P‐doped carbon coated bimetallic CoMo carbides and oxides nanoparticles that are highly dispersed on N,P‐doped reduced graphene oxide (NPRGO). To this end, we used Keggin polyoxomolybdates [H_3_PMo_12_O_40_] (=**PMo_12_**) and Co(OAc)_2_ **⋅**4 H_2_O as the Mo and Co precursors, respectively, and the melamine as the carbon source and graphene oxide (GO) as the conducting support for the construction of well dispersed bimetallic CoMo carbides and oxides, based on the following considerations: 1) The **PMo_12_** serves as molecular precursor with abundant Mo atoms and can be dissolved in water. 2) The melamine can be grafted to GO through multiple interactions including covalent interactions (between carboxyl groups in GO and amine groups in melamine), hydrogen bonding (between amine groups in melamine and oxygen‐containing groups in GO) and π–π interactions (between GO and triazine rings of melamine), thus forming a stable GO‐melamine complex.^38^ 3) The hydrogen bonding and the electrostatic interaction between **PMo_12_** and melamine further benefits the high dispersion of **PMo_12_**. Interestingly, we note that different crystalline structures can be obtained by adjusting the Mo/Co molar ratio, and the composites of N,P‐doped carbon coated bimetallic Co_6_Mo_6_C_2_/Co_2_Mo_3_O_8_ nanoparticles decorated on N,P‐doped reduced graphene oxide (Co_6_Mo_6_C_2_/Co_2_Mo_3_O_8_/NPCRGO) obtained at the optimized Mo/Co molar ratio of 3:1 (composite **4**) shows superior electrocatalytic activity for full water splitting in alkaline conditions.

## Results and Discussion

### Synthesis

**Scheme 1:**
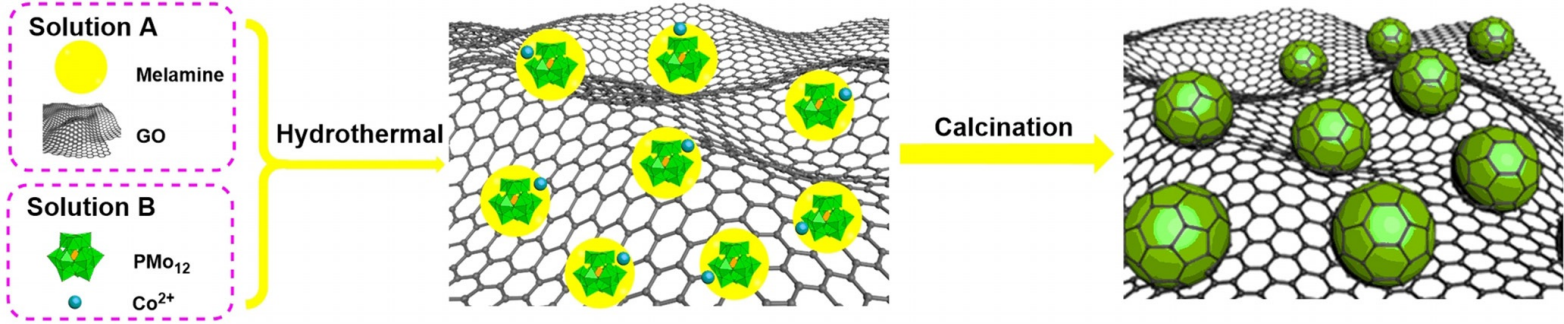
Schematic illustration of the synthesis of the bimetallic CoMo carbides/oxide/NPCRGO composites.

Scheme 1 shows the synthetic process for the composite materials. During synthesis, two solutions were prepared. For Solution A, melamine and graphene oxide (GO) were dispersed in water by vigorous stirring, forming a stable GO–melamine dispersion. For Solution B, **PMo_12_** and Co(OAc)_2_ were dissolved in water, for details see the Experimental Section. Next, both solutions were combined to give a homogeneous dispersion. Hydrothermal reaction of this solution results in an initial composite which was dried and carbonized in argon, forming bimetallic CoMo carbides/oxides with NPCRGO composites. Note that different bimetallic CoMo carbides/oxides (Co_6_Mo_6_C_2_, Co_2_Mo_3_O_8_, CoMoO_4_) can be accessed easily by controlling the Mo/Co molar ratios (1:1, 2:1 and 3:1), which will be demonstrated later. The products obtained for Mo/Co molar ratios of 1:1, 2:1 and 3:1 were named as composites **2**, **3**, and **4**, respectively. Interestingly, composite **4** contains only Co_6_Mo_6_C_2_ and Co_2_Mo_3_O_8_ as crystalline phases, and shows the best performance for both OER and HER, whereas composites **2** and **3** contain mixed species of Co_6_Mo_6_C_2_, Co_2_Mo_3_O_8_, CoMoO_4_, and Mo_2_C. For comparison, pure PMo_12_ without Co^2+^ was also used for the preparation of the reference sample of composite **1**.

### Characterization

**Figure 1:**
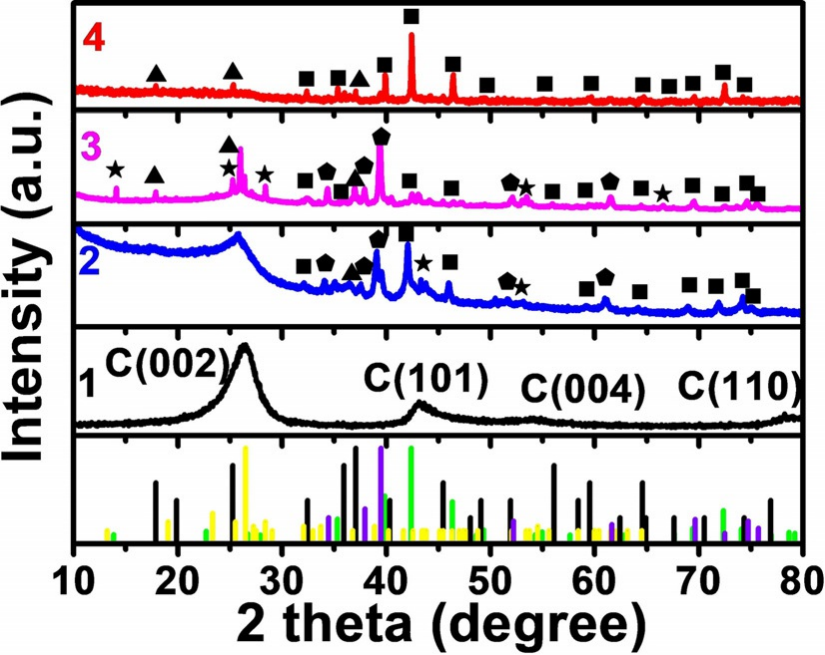
XRD analysis of composites **1**–**4**. The symbols are shown as: square: (Co_6_Mo_6_C_2_, JCPDS # 80‐0339, green line), triangle: (Co_2_Mo_3_O_8_, JCPDS # 34‐0511, black line), star: (CoMoO_4_, JCPDS # 21‐0868, yellow line), and pentagon: (Mo_2_C, JCPDS # 11‐0680, purple line).

pXRD was used to analyze the chemical structures of the composites prepared at different Mo/Co molar ratio (Figure 1). For catalyst **1**, only peaks for graphitic carbon can be seen, while no crystalline features for metal oxides or carbides are observed. When Co^2+^ is present in the system (i.e. samples **2**, **3**, **4**, Figure 1), we note significant changes of the composition. For both catalysts **2** and **3**, it is shown that mixtures of Co_6_Mo_6_C_2_, Co_2_Mo_3_O_8_, CoMoO_4_, and Mo_2_C are present: in **2**, Co_6_Mo_6_C_2_ forms the main crystalline species, whereas in **3**, Mo_2_C is the main species. In contrast, for **4**, Co_6_Mo_6_C_2_ was the dominant phase with only small contributions from Co_2_Mo_3_O_8_. The result shows that the Mo/Co molar ratio plays an important role in the forming of bimetallic Co_6_Mo_6_C_2_.

Next, aberration‐corrected high‐resolution TEM (AC‐HRTEM) were performed to examine the morphologic structure of the composites. For composite **1**, we can see clearly the wrinkle structure of graphene with the large scale image shown in Figure S1a, Supporting Information. The HRTEM image shown in Figure S1b indicates the typical stacked structures of few layer‐graphene and also the graphitic carbon can be seen, but not any crystalline nanoparticles, which agrees well with the XRD data.

**Figure 2:**
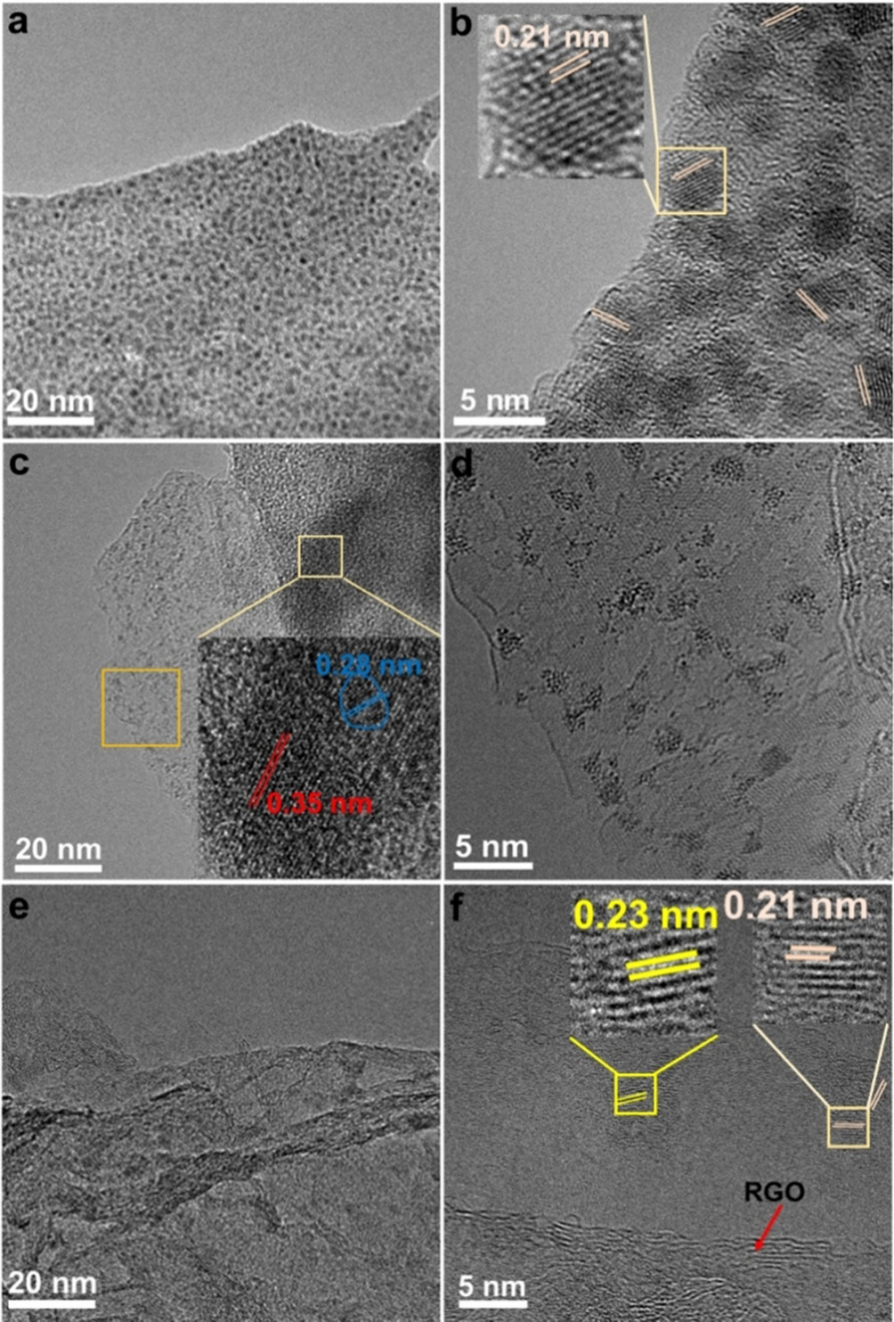
TEM overview and aberration corrected‐HRTEM images of **2** (a, b), **3** (c, d) and **4** (e, f).

**Figure 3:**
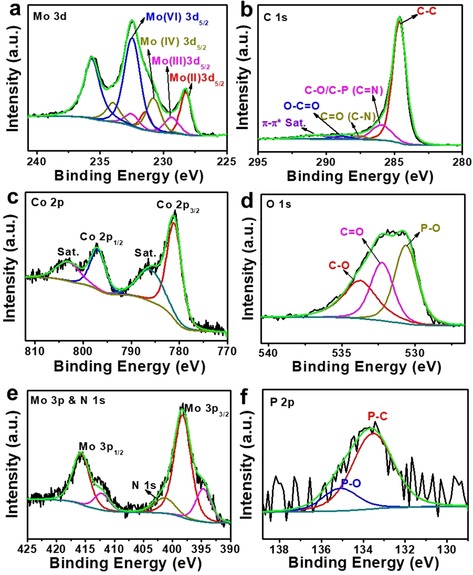
HRXPS analysis of **4**: (a) Mo 3d, (b) C 1 s, (c) Co 2p, (d) O 1 s, (e) mixed Mo 3p and N 1 s, (f) P 2p.

Figure 2 a and b show the different magnifications of composite **2**, Figure 2 c and d show the different magnifications of composite **3**, and Figure 2 e and f show the different magnifications of composite **4**. For **2** and **4**, both composites show the high dispersion of the main species of Co_6_Mo_6_C_2_ nanoparticles (the lattice spaces of 0.21 and 0.23 nm were designated to the crystal faces of Co_6_Mo_6_C_2_) decorated on the surface of the RGO film. The Co_6_Mo_6_C_2_ nanoparticles differ in size: for **4**, we observe average particles size of 2.0±0.2 nm. Whereas for composite **2**, we observe an average particle size of 3.0±0.5 nm. Particle sizes were determined from TEM images. In comparison, we also saw a few large Co_2_Mo_3_O_8_ particles besides the small Co_6_Mo_6_C_2_ particles in composite **2** (Figure S1c, Supporting Information). Moreover, thinner graphene films can be also observed in **4** compared with **2**, indicating the GO was reduced more thoroughly, which can enhance the conductivity and thus the catalytic activity of the sample. However, significantly different observations can be found for composite **3**. As shown in the insert of Figure 3 c (a magnified image corresponding to the gold square area), we observed both small particles (the lattice spacing of 0.28 nm is in line with Co_6_Mo_6_C_2_ (400), CoMoO_4_ (−131) and Co_2_Mo_3_O_8_ (103)) and large single crystalline particles (the lattice spacing of 0.35 nm can be assigned to CoMoO_4_ (201) and Co_2_Mo_3_O_8_ (102)). Besides, many small clusters with the size of 0.1–2.0 nm can be also seen (Figure 3 d, a magnified image corresponding to the orange square area shown in Figure 3 c) that are supported on the few layer graphene structures (some moiré patterns were formed due to the stacked structures of few layer‐graphene in composite **3**, Figure S1d). Finally, it should be noted that all the lattice fringes observed by TEM are in agreement with the pXRD data.

In sum, these analyses show that by variation of the Mo/Co molar ratio, we can tune the formation of different mixed‐metal oxide/carbide phases and trigger their deposition as ultrasmall nanoparticles with uniform dispersion on conductive carbon substrates.

XPS was used to assess the elemental composition and chemical state of the composites. Figure S2a and Figure 3 show the XPS for composite **4**. The survey XPS spectrum of **4** shows the presence of Co, Mo, O, C, and N (Figure S2a, Supporting Information). Figure 3 a shows the high‐resolution deconvoluted Mo 3d spectrum, indicating the presence of Mo in four oxidation states (+2, +3, +4, and +6). Oxidation states +2 and +3 are characteristic for Mo–carbide species, while the +4 oxidation state can be assigned to the Co_2_Mo_3_O_8_ particles and possibly to surface oxidation of the bimetallic carbides. The +6 oxidation state species are assigned to possible surface oxidation of both the bimetallic carbides and oxides.^26^, ^34^ Figure 3 b shows the deconvoluted C 1 s spectrum, with four peaks assigned to C−C, C−O/C−P/C=N, C=O/C−N, and O−C=O species, indicating that P and N are doped into the carbon framework. Figure 3 c shows the deconvoluted Co 2p spectrum which is dominated by Co^2+^ species. The O 1 s spectrum shows that three functional groups, P−O, C=O, and C−O, are observed (Figure 3 d).

The Mo 3p region shown in Figure 3 e also contains the N 1 s peak that can be split into pyridinic N, pyrrolic N and graphitic N (Figure S2b, Supporting Information), indicating that N was also doped into the carbon. The deconvoluted P 2p spectrum shows two peaks, assigned to P−C and P−O bonds (Figure 3 f). In sum, C 1s, N 1s, P 2p and O 1s analyses suggest the N‐ and P‐ doping of the carbon matrix. Based on the above analyses, catalyst **4** can be described as bimetallic Co_6_Mo_6_C_2_ and Co_2_Mo_3_O_8_ nanoparticles deposited on N, P‐doped reduced graphene oxide (Co_6_Mo_6_C_2_/Co_2_Mo_3_O_8_/NPCRGO). The XPS analysis of composites **2** and **3** are discussed in the Supporting Information (Figures S3 and S4) for brevity. Note that when comparing composites **2**–**4**, composite **4** has the highest Mo^2+^/Mo^3+^ molar ratio (1.6:1), which is favorable to boost the HER activity.^26^ Moreover, in **4**, pyridinic N is the dominant phase (Table S1, Supporting Information), which is considered beneficial for electrochemical process.^39^ All XPS data are summarized in the Supporting Information, Table S2.

### Electrocatalytic HER studies

**Figure 4:**
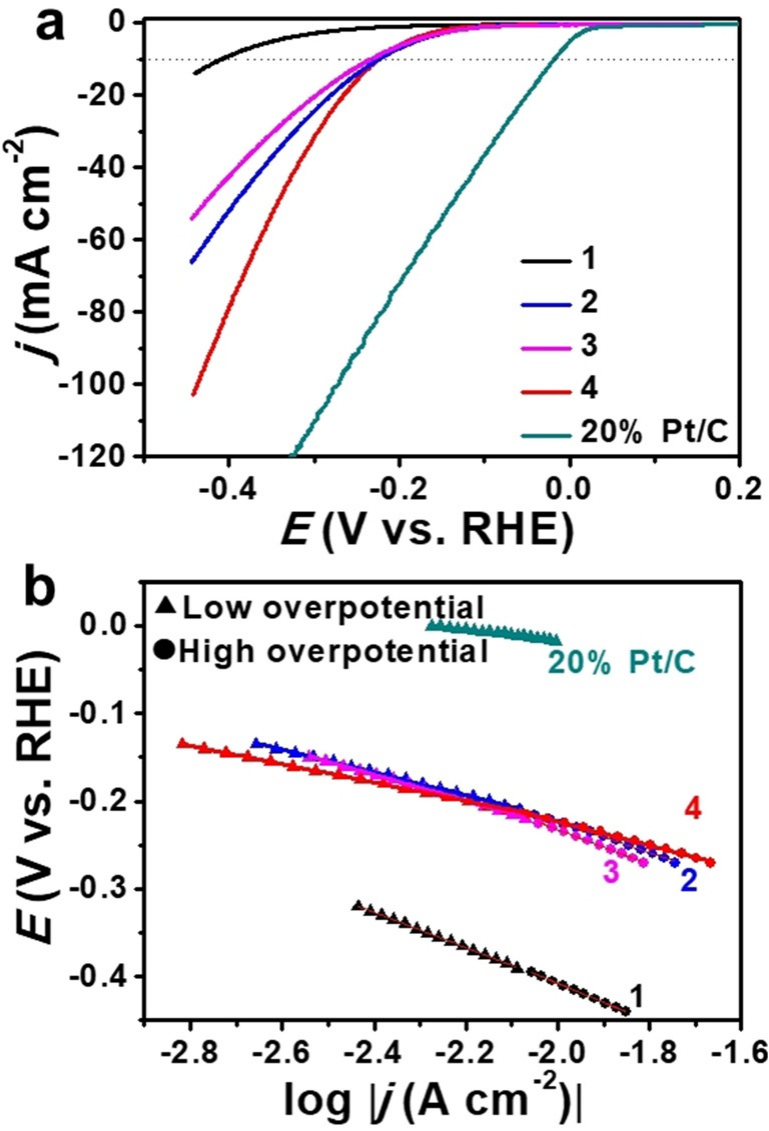
Electrocatalytic HER performance. (a) LSV curves of **1**–**4** and 20 wt‐% Pt/C modified rotating disk electrode (RDE) in 1 m aqueous KOH at a sweep rate of 5 mV s^−1^ and rotating speed of 1600 rpm. (b) The corresponding Tafel plots derived from the LSV data, note that both the low and high overpotential regions were used for Tafel analyses.

Based on the promising combination of ultrasmall metal carbide/oxide particles, high particle dispersion and high conductivity of the rGO carbon matrix, we then explored the composite performance in both HER and OER. First, we studied the electrocatalytic HER activity of the catalysts (Figure 4). The catalysts were dispersed in EtOH and drop‐cast on the rotating disk electrode (RDE, the loading amount was 0.8 mg cm^−2^). Figure 4 a shows the iR‐corrected linear sweep voltammetric (LSV) curves of different catalysts modified RDE in 1 m aqueous KOH. As a reference point for performance comparison, we used the overpotential at a current density of 10 mA cm^−2^ (η_10_). Our data shows that **2** (223 mV) and **4** (220 mV) feature similar η_10_ values, whereas **3** showed increased overpotentials of 233 mV. The carbide/oxide‐free reference **1** showed the largest η_10_ value (408 mV). With increasing current densities >10 mA cm^−2^, we note that composite **4** shows increasing HER activity, lower overpotentials were needed for **4** than **2** when the current density goes larger than 10 mA cm^−2^, indicating the improving performance of **4** at higher current densities. As described above based on XRD and TEM analysis, bimetallic Co_6_Mo_6_C_2_ was the main species in **2** and **4**, which suggests that the crystalline bimetallic Co_6_Mo_6_C_2_ could be the main active site for HER, and the dual N,P‐doping in the carbon further increases the activity. Notably, composite **4** shows higher HER activity compared with related literature examples of mixed Mo–Co sulfides with lower η_10_; however, care should be taken in comparison, as these literature examples were measured in different electrolytes at different pH.^40^, ^41^ Tafel slope analysis (Figure 4 b) was investigated in both the low and high overpotential range (Tafel slope values are shown in Table 1). Composite **4** gives the lowest Tafel slopes in both the low and high overpotential range (104.7±0.4 and 136.0±1.9 mV dec^−1^), indicating the fast HER kinetics of **4**. The values show that the HER kinetics for all the prepared catalysts may be decided by the Volmer–Heyrovsky step.^2^ For comparison, 20 % Pt/C (η_10_ of 17 mV and Tafel slope of 62.9±1.0 mV dec^−1^) was also recorded.

**Table 1:** Comparison of the electrocatalytic HER and OER performance of different catalysts.

Samples	Overpotential [mV]^[a]^		Tafel slopes [mV dec^−1]^		ECSA [cm^2][b]^	*R*_ct_ [Ohm]
	HER	OER	HER	OER		
**1**	408	556	201.4±0.7^[c]^ 217.4±1.3^[d]^	228.4±3.4	–	–
**2**	223	441	130.2±1.2^[c]^ 177.1±2.3^[d]^	104.3±1.2	852.8±5.0	3.8
**3**	233	>700	147.8±1.5^[c]^ 198.3±2.0^[d]^	531.4±6.0	719.3±3.3	1.5
**4**	220	403	104.7±0.4^[c]^ 136.0±1.9^[d]^	89.7±0.8	1025.3±4.8	1.0
IrO_2_	–	409	–	90.5±2.4	–	–
20 % Pt/C	17	–	62.9±1.0	‐	–	–

[a] Overpotential η_10_ at *j*=10 mA cm^‐2^. [b] For details of ECSA calculation, see the Experimental Section. [c] Tafel slope in low overpotential range. [d] Tafel slope in high overpotential range.

### Electrocatalytic OER studies

**Figure 5:**
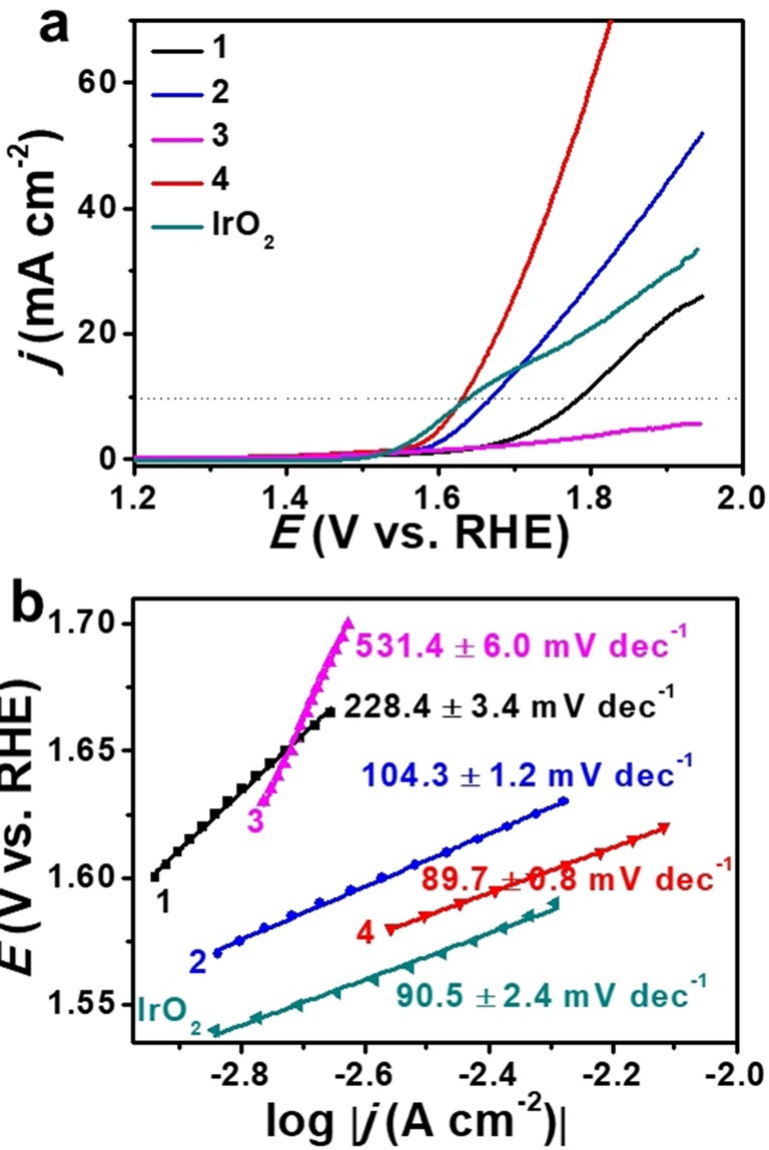
Electrocatalytic OER performance. (a) LSV curves of **1**, **2**, **3**, **4** and IrO_2_ modified rotating disk electrode (RDE) in 1 m aqueous KOH at a sweep rate of 5 mV s^−1^ and rotating speed of 1600 rpm. (b) The corresponding Tafel plots derived from the LSV data, note that the low overpotential region was used for Tafel analyses.

Next, composites **1**–**4** were also studied for electrocatalytic OER in 1 m aqueous KOH. Figure 5 a shows the polarization curves for the catalysts, and the commercial reference compound IrO_2_. Under the given conditions, **4** features the highest electrocatalytic OER activity with the lowest η_10_ of 403 mV, while catalysts IrO_2_, **1**, **2** and **3** showed higher overpotentials of 409, 556, 441, and >700 mV, respectively. Notably, composite **3**, which features Mo_2_C as the main component, shows the lowest OER activity, highlighting that this monometallic carbide is not an active OER catalyst, which is in line with literature.^19^ Tafel slope analysis (Figure 5 b) in the low overpotential range shows that **4** features the lowest Tafel slope amongst the catalysts tested, indicating the fast OER kinetics of **4**. For detailed comparison, see Table 1.

### Electrocatalytic overall water splitting

**Figure 6:**
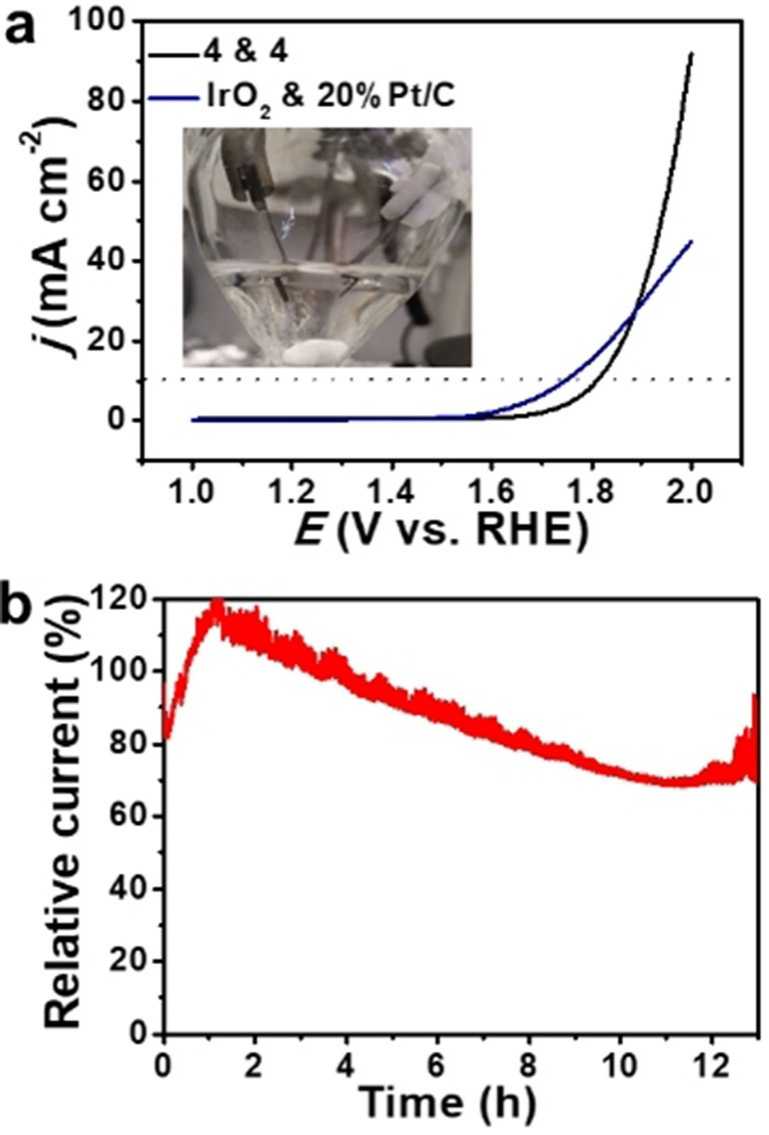
Electrocatalytic full water electrolysis. (a) LSV curves of system **4** (**4**‐cathode + **4**‐anode) and the IrO_2_ & 20 % Pt/C reference system. The insert shows the experimental set‐up. (b) Chronoamperometry of the system **4** at *E=*1.85 V. Conditions: Catalysts were deposited on carbon paper, electrolyte: 1 m aqueous KOH.

Finally, we chose composite **4** to assemble a full water‐splitting cell (2‐electrode setup) by using **4** as cathode and anode in an alkaline water electrolyzer. To this end, **4** was loaded on pre‐treated carbon paper (geometric area: 1 cm^2^, the treatment of carbon paper is given in the Experimental Section) with a loading amount of 0.5 mg cm^−2^ for both anode and cathode. As a reference, commercial 20 % Pt/C and IrO_2_ were deposited on carbon paper in an identical fashion and used as cathode and anode for water electrolysis, respectively. Figure 6 a shows the full water electrolysis performance for both systems (the insert shows the experimental setup for the electrolysis). For the system based on the **4**‐cathode and **4**‐anode, a water‐splitting current density of 10 mA cm^−2^ was achieved at a cell voltage of 1.81 V. Note that the commercial reference system using Pt/C and IrO2 reached 10 mA cm^−2^ at a slightly lower cell voltage (1.75 V). Notably, at higher current densities >25 mA cm^−2^, the **4**‐based electrolyzer showed better performance compared with the PtC/IrO_2_ system (Figure 6 a), which could be attributed to the faster kinetics in the high overpotential range, demonstrating the potential of the noble‐metal‐free system **4**. The long‐term stability of **4** for full water splitting was evaluated by chronoamperometry at *E=*1.85 V (Figure 6 b). Over the course of the 12 h test, we note an initial conditioning phase, where a brief drop in activity (≈20 %, *t=*0–1 h) is followed by a catalytic activity increase to ≈120 % (*t=*1–2 h). After this, the system shows decreasing current densities (*t=*2–10 h) which then stabilize at ≈70 % after 11 h of operation. Furthermore, the structural stability of composite **4** after water splitting was also investigated by scanning electron microscopy (SEM) and corresponding energy‐dispersive X‐ray spectroscopy (EDS) mapping, Raman spectroscopy and XPS analyses (Figures S5 to S11, Supporting Information). These post‐catalytic analyses of composite **4** show no significant structural or elemental changes after water splitting in 1 m KOH. Next, we investigated the Faradaic efficiency of **4** for both HER and OER at *j*=20 mA cm^−2^. To this end, the gas evolution (H_2_ and O_2_, respectively) was assessed volumetrically using a custom‐built setup (Figure S12a, Supporting Information). For both catalytic reactions, the Faradaic efficiencies reached ≈100 % (Figure S12b, Supporting Information), and the experimentally determined H_2_ volume was twice the amount of the generated O_2_, which agrees with the reaction stoichiometry (Figure S12c, Supporting Information).

In order to gain initial mechanistic insights into the superior water‐splitting performance of **4**, we determined the electrochemically active surface area (ECSA) and the charge transfer resistance (*R*_ct_) at the electrode/electrolyte interface, which are key parameters affecting the reactivity of heterogeneous electrocatalysts. For this purpose, we first performed electrochemical double‐layer capacitance analyses (*C*_dl_, Figure S13, Supporting Information) to obtain the ECSA values of **2**, **3** and **4** 42, 43 As summarized in Table 1, we observe significantly higher ECSA values for **4** compared with **2** and **3**, which is in the order of **4**>**2**>**3**. We note that composites **2** and **4**, which contain Co_6_Mo_6_C_2_ as the main chemical species, show the highest ECSA values. The correlation between these two factors is currently under investigation. Further, electrochemical impedance spectroscopy (EIS) analysis shows that **4** features the lowest *R*_ct_ (Figure S14 and Table 1), indicating that in **4**, efficient and fast electron transfer at the solid‐electrolyte interface is possible.^29^

## Conclusions

In conclusion, we have reported a rational design method for the synthesis of bimetallic metal carbide/oxide nanoparticles deposited on reduced graphene oxide matrices. The resulting composites show promising performance as bifunctional catalysts for the hydrogen evolution reaction and the oxygen evolution reaction. Full electrochemical water splitting was possible by using the identical composite electrodes as both cathode and anode, and remarkable performance similar to noble‐metal‐based references was observed. Stability analyses show that after an initial loss of activity, stable performance at ≈70 % of the initial current density is possible, suggesting that materials optimization concepts could be used to improve this performance for enhanced technological relevance. This work therefore provides a rational materials–design approach which gives access to mixed‐metal carbides/oxides as (electro‐)catalysts for energy‐relevant multielectron reactions.

## Experimental Section

### Preparation of the catalysts

Briefly, 20 mL melamine (Alfa Aesar) solution (2 mg mL^−1^ in aqueous phase) was first mixed with 20 mL GO solution (4 mg mL^−1^ in aqueous phase, prepared by a modified Hummers method) by stirring at 700 rpm for 15 min. Then, phosphomolybdic acid hydrate (27.4 mg, 0.015 mmol [H_3_PMo_12_O_40_]**⋅** *x* H_2_O (=PMo_12_), Alfa Aesar) and Cobalt(II) acetate tetrahydrate (Co(OAc)_2_ **⋅**4 H_2_O, Merck, containing 44.8, 22.4 and 14.9 mg (0.18, 0.09 and 0.06 mmol) for composite **2**, **3** and **4**) were dissolved in 40 mL H_2_O and added to the above mixture under stirring at 1000 rpm for 6 h. Afterwards, the obtained mixtures were hydrothermally reacted at 180 °C for 12 h, and then cooled down to room temperature. The obtained solid composites were filtered off, washed with water for three times, and dried at 80 °C for overnight. Finally, the dried composites were calcined in the tube furnace programmed with two heating steps under Ar atmosphere, first at 400 °C for 2 h with a heating rate of 1 °C min^−1^, and then at 800 °C for 2 h with a heating rate of 2 °C min^−1^. This gave the final composites **2**, **3**, and **4** with the Mo/Co molar ratio of 1:1, 2:1 and 3:1, respectively. For comparison, we also prepared the catalyst **1** without adding Co(OAc)_2_ **⋅**4 H_2_O, and the other conditions were kept the same.

### Characterization

TEM measurements were performed using an image‐side aberration corrected FEI Titan 80–300 at 80 kV accelerating voltage. The samples were drop‐casted on holey carbon grids prior to the TEM investigations. PXRD studies were performed on a BRUKER D8 Advance XRD unit using Cu_Kα_ (*λ*=1.54 Å). XPS analysis was performed on ESCALAB250 Thermo Electron Corporation equipment with an Al Ka X‐ray source (1486.6 eV). The X‐ray source was run at a reduced power of 150 W, and the pressure in the analysis chamber was maintained at <10^−11^ Pa.

### Electrocatalytic HER and OER

5 mg of the finely ground catalyst (**1**, **2**, **3**, **4**, or 20 % Pt/C) was dispersed in 980 μL anhydrous ethanol containing 20 μL 5 % Nafion solution ([catalyst]=5 mg mL^−1^) by sonication for 1 h to form a homogeneous ink. Then, 20 μL of the catalyst ink were dropped onto a glassy carbon (GC) rotating disk electrode (RDE) with 4 mm diameter (the loading amount was 0.8 mg cm^−2^). After drying, the electrodes were further modified with a thin film of Nafion by dropping 1.0 μL 0.5 wt % Nafion solution (in isopropanol) onto the electrode surface. A standard three‐electrode cell was used and was operated at room temperature. The prepared thin‐film covered RDE was used as the working electrode. Glassy carbon rod (for HER) or platinum foil (for OER) was used as counter electrode and an Hg/HgO (1 m KOH) electrode was used as reference electrode. The electrode was performed 50 CV cycling beforehand for stabilization. HER and OER measurements were performed on a CHI 730E electrochemical system (CH Instruments Inc.). EIS experiments were performed in the same electrolyte on a CHI 760E electrochemical system (CH Instruments Inc.) in the frequency range from 1000 kHz to 0.01 Hz with modulation amplitude of 5 mV.

The Hg/HgO electrode was referenced against the reversible hydrogen electrode (RHE) in all measurements. The referencing was performed based on the Nernst equation [Eq. 1]:

$$E{}_{\mathrm{RHE}}=E{}_{\mathrm{Hg}/\mathrm{HgO}}+E{}^{0}{}_{\mathrm{Hg}/\mathrm{HgO}}+0.059\;\mathrm{pH}.$$

For 1 m aqueous KOH [Eq. 2]:

$$E{}_{\mathrm{RHE}}=E{}_{\mathrm{Hg}/\mathrm{HgO}}+0.945\;V.$$

ECSA values are calculated based on the equations [Eqs. 3–5] shown below:

$$\Delta j=\gamma \times C{}_{\mathrm{dl}}$$

$$\mathrm{ECSA}=C{}_{\mathrm{dl}}/C{}_{s}$$

$$C{}_{s}=40\;\mu F\;\mathrm{cm}{}^{-2}\;\mathrm{per}\;\mathrm{cm}{}^{2}$$

in which *γ* is the scanning rate, *Δj* (=*j*_a_−*j*_c_) is the charging current density differences, *C*_dl_ is the double‐layer capacitance, and *C*_s_ is the specific capacitance of the catalyst.

### Electrocatalytic overall water splitting

The carbon paper was used as the catalyst support during water electrolysis, which was washed and sonicated in acetone, ethanol and water for 20 min each before used. For preparing the electrodes for water electrolyzer, 100 μL of the catalyst **4** ink were separately dropped onto two pre‐treated carbon papers with geometric surface area of 1 cm^2^ (the loading amount was 0.5 mg cm^−2^). After drying, the electrodes were further modified with a thin film of Nafion by dropping 10 μL 0.5 wt % Nafion solution (in iso‐propanol) onto the electrode surface. These two **4**‐modified carbon papers were used as anode and cathode in the water electrolyzer in 2‐electrode setup. As a reference, 20 % Pt/C and IrO_2_ (the IrO_2_ ink was prepared following the same procedure as other catalysts) were assembled into the water electrolyzer as cathode and anode, respectively, with the same catalyst loading amount as **4**.

## Conflict of interest

The authors declare no conflict of interest.

## Supporting information

As a service to our authors and readers, this journal provides supporting information supplied by the authors. Such materials are peer reviewed and may be re‐organized for online delivery, but are not copy‐edited or typeset. Technical support issues arising from supporting information (other than missing files) should be addressed to the authors.

Supplementary
