# Supplementary material for: Bottom‐up Design of Bimetallic Cobalt–Molybdenum Carbides/Oxides for Overall Water Splitting
Source: Chemistry. 2020 Jan 30;26(18):4157–64. doi: 10.1002/chem.201905265 (PMC7154525; doi:10.1002/chem.201905265)
Supplement: Supplementary file 1 — Supplementary [file CHEM-26-4157-s001.pdf]

CHEMISTRY
A European Journal

Supporting Information

Bottom-up Design of Bimetallic Cobalt–Molybdenum Carbides/ Oxides for Overall Water Splitting

Rongji Liu,*[a, b, c] Montaha Anjass,[a, d] Simon Greiner,[a, d] Si Liu,[a] Dandan Gao,[a] Johannes Biskupek,[e] Ute Kaiser,[d, e] Guangjin Zhang,[b, c] and Carsten Streb*[a, d]

chem_201905265_sm_miscellaneous_information.pdf

Experimental Methods

TEM and HRTEM measurements were performed using an image-side aberration corrected FEI Titan 80-300 at 80 kV accelerating voltage. The samples were drop-cast on holey carbon grids prior to the TEM investigations..

The scanning electron microscopy (**SEM**) and Energy-dispersive X-ray spectroscopy (**EDS**) were performed on a Zeiss Gemini LEO 1550 VP equipped with a Silicon Drift Detector (OXFORD Instruments) using a 10 kV electron beam. The HAADF-STEM and EDS was performed using a probe-corrected JEOL JEM-ARM200F TEM/STEM at 100 kV accelerating voltage. This system was also used to acquire local EDS mapping.

PXRD studies were performed on a BRUKER D8 Advance XRD unit using Cu−Kα (λ = 1.54 Å).

XPS analysis was performed on ESCALAB250 Thermo Electron Corporation equipment with an Al Ka X-ray source (1486.6 eV). The X-ray source was run at a reduced power of 150 W, and the pressure in the analysis chamber was maintained at $<10^{-11}$ Pa.

Electrochemical experiments were performed on a CHI 760E electrochemical system (CH Instruments Inc.).

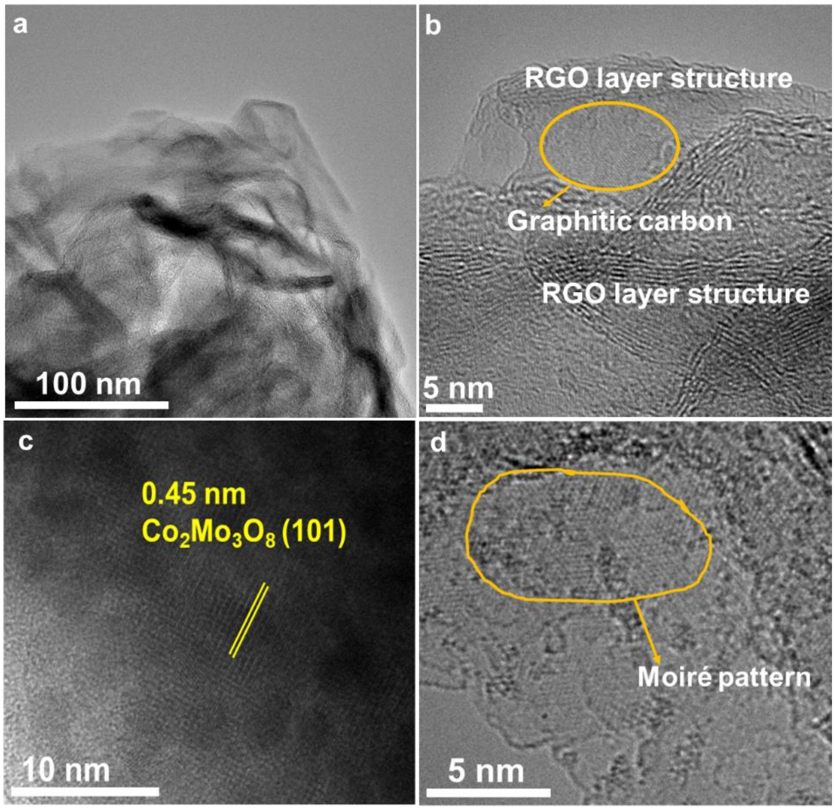

Figure S1. (a) Overview image of composite **1**. (b) AC-HRTEM image showing details of the carbon support that contain graphitic and layered carbon. (c) AC-HRTEM image of composite **2** showing large $Co_2Mo_3O_8$ particles. (d) The moiré pattern formed in the stacked structures of few layer-graphene in composite **3**.

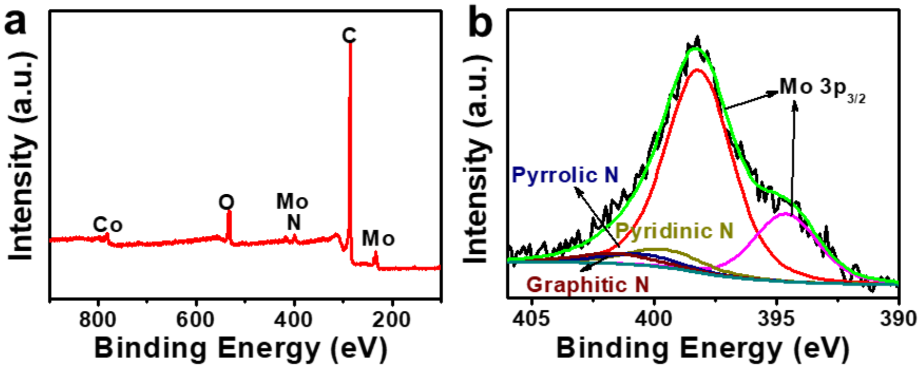

Figure S2. (a) Survey XPS spectrum of **4**. (b) deconvoluted N 1s .

Table S1. Atomic contents of different elements from XPS analysis

Samples	**Atomic content of different types of N doping (%)**		
	Pyridinic N	Pyrrolic N	Graphitic N
**2**	0	0	0
**3**	0.59	0.72	0.56
**4**	0.27	0.17	0.17

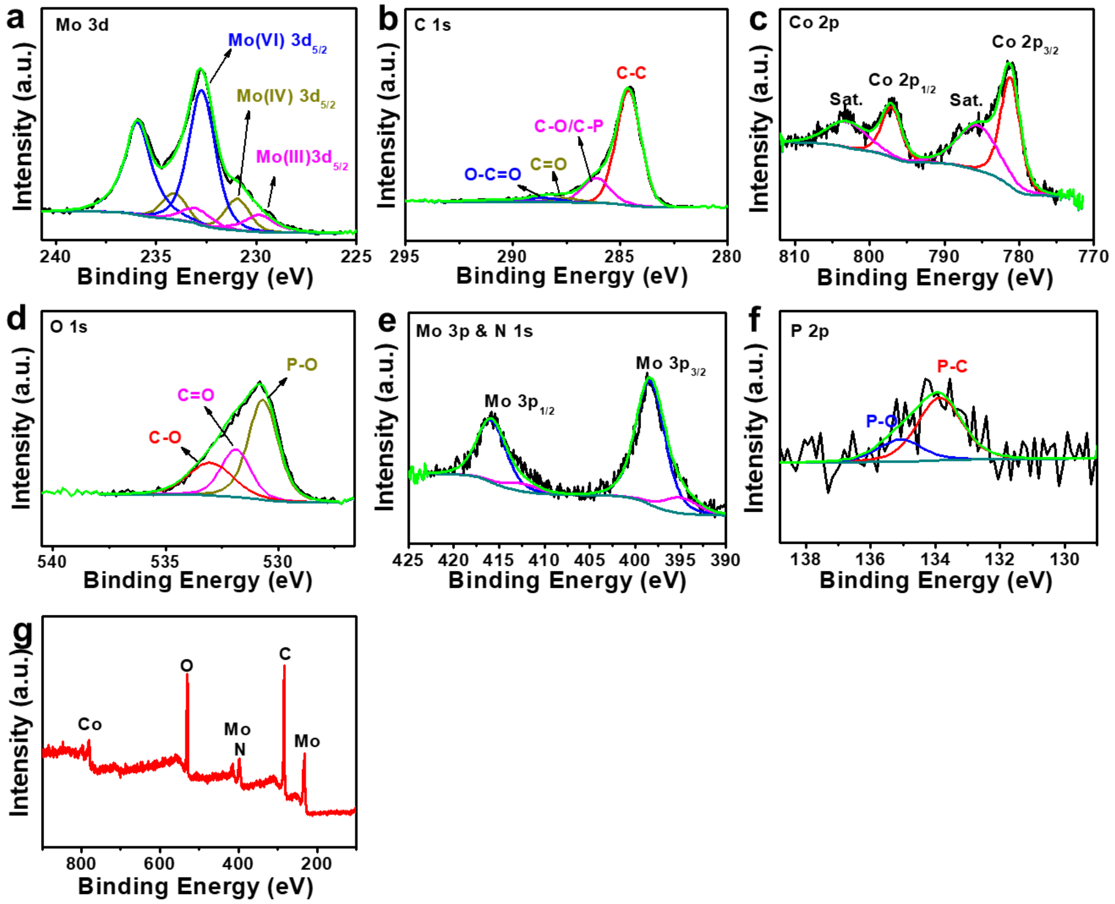

Figure S3. High resolution XPS analysis of **2**. (a) Mo 3d, (b) C 1s, (c) Co 2p, (d) O 1s, (e) mixed Mo 3p and N 1s, (f) P 2p. (g) Survey XPS spectrum of **2**.

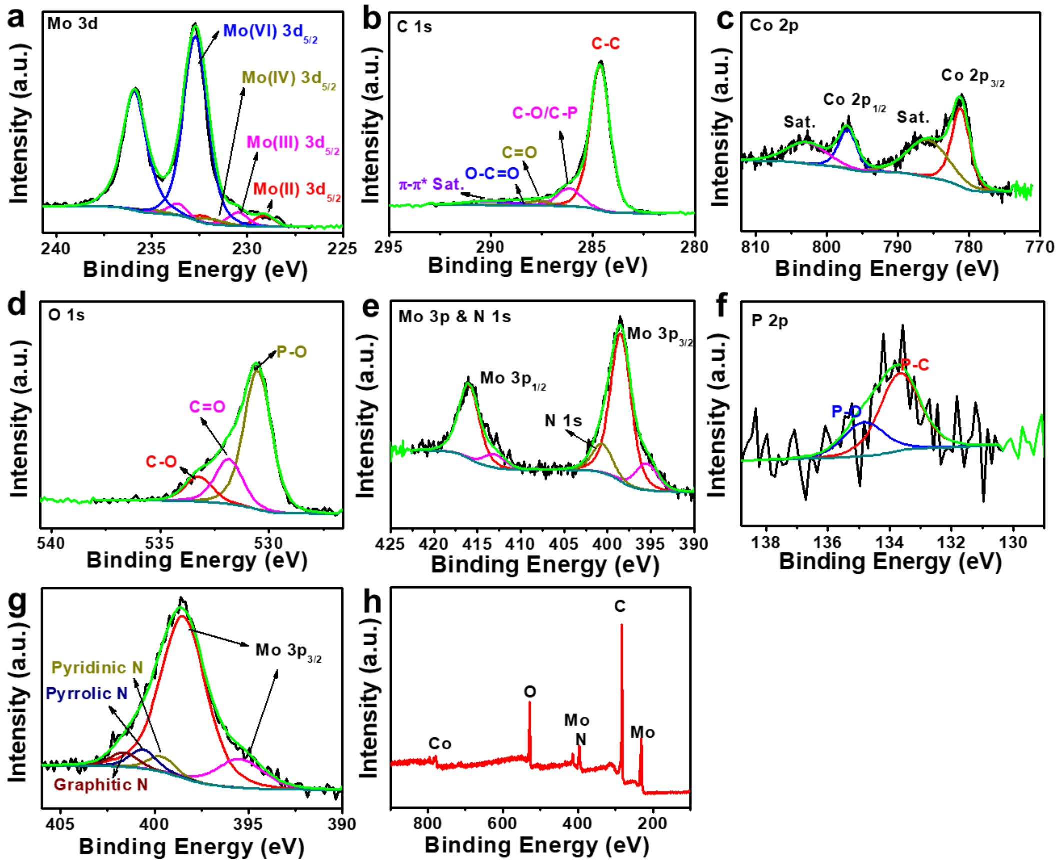

Figure S4. High resolution XPS analysis of **3**. (a) Mo 3d, (b) C 1s, (c) Co 2p, (d) O 1s, (e) mixed Mo 3p and N 1s, (f) P 2p, (g) deconvoluted N 1s. (h) Survey XPS spectrum of **3**.

Table S2. Binding energies of different elements from XPS analysis

Samples		Composite **2**	Composite **3**	Composite **4**
Mo 3d	Mo^{II} 3d $_{5/2}$	-	229.1	228.3
	Mo^{II} 3d $_{3/2}$	-	232.3	231.4
	Mo^{III} 3d $_{5/2}$	229.8	230.5	229.3
	Mo^{III} 3d $_{3/2}$	233.0	233.6	232.5
	Mo^{IV} 3d $_{5/2}$	231.0	231.9	230.8
	Mo^{IV} 3d $_{3/2}$	234.1	235.1	234.0
	Mo^{VI} 3d $_{5/2}$	232.7	232.7	232.5
	Mo^{VI} 3d $_{3/2}$	235.9	235.9	235.7
P 2p	P-C	133.9	133.7	133.3
	P-O	135.1	134.8	134.9
C 1s	C-C	284.6	284.7	284.6
	C-O/C-P (/C=N)	286.1	286.2	285.9
	C=O (/C-N)	287.6	287.7	287.6
	O-C=O	288.6	288.8	288.8
O 1s	P-O	530.7	530.5	530.6
	C=O	531.9	531.9	532.3
	C-O	533.0	533.2	533.7
N 1s	Pyridinic N	-	399.6	399.6
	Pyrrolic N	-	400.6	400.6
	Graphitic N	-	401.6	401.6
Co 2p	Co 2p $_{3/2}$	781.2	781.2	780.9
	Co 2p $_{1/2}$	797.0	797.1	796.7

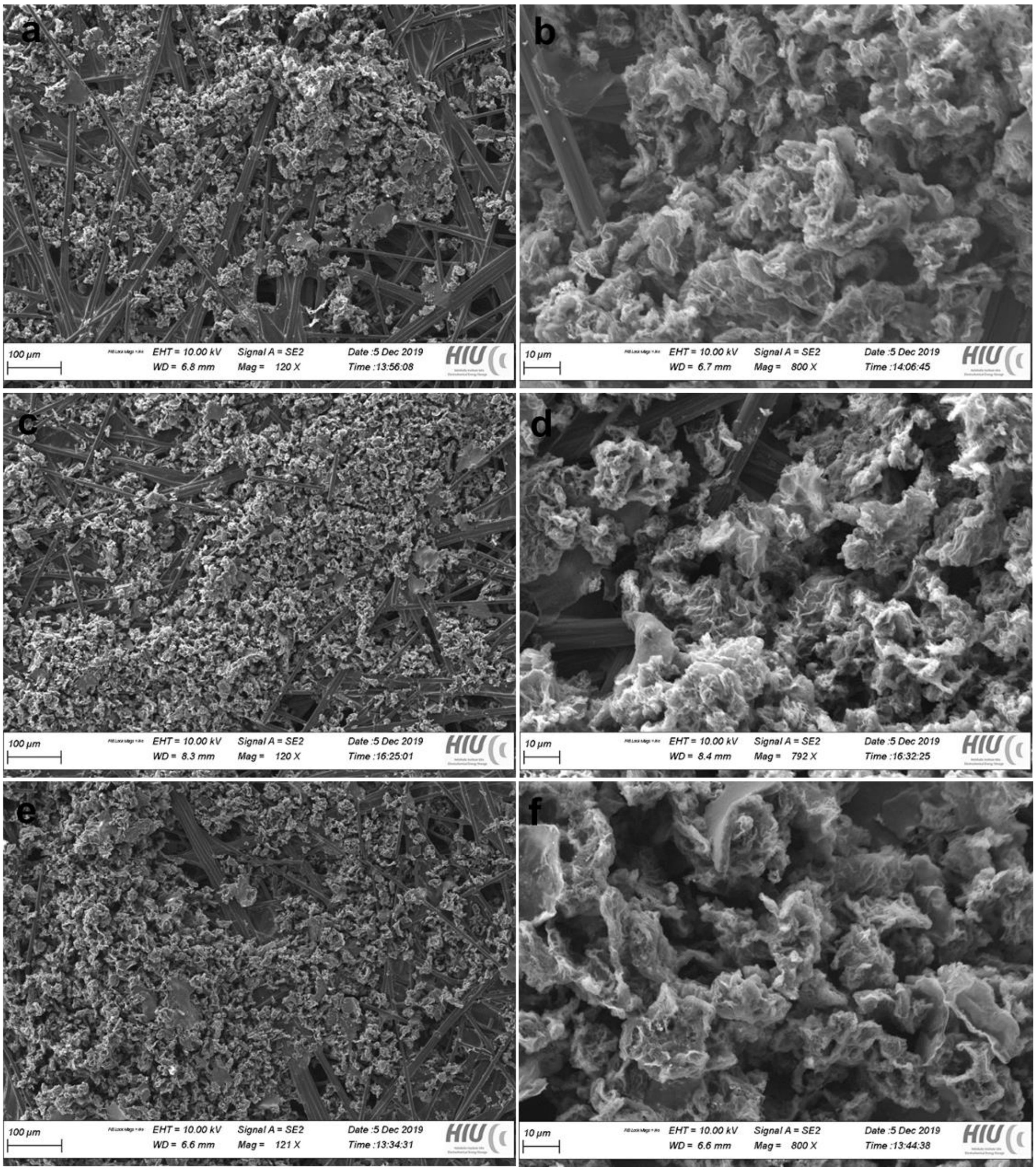

Figure S5. SEM images of composite **4** modified carbon paper before and after water splitting. (a, b) before water splitting. (c, d) after water splitting on the anode (after OER). (e, f) after water splitting on the cathode (after HER).

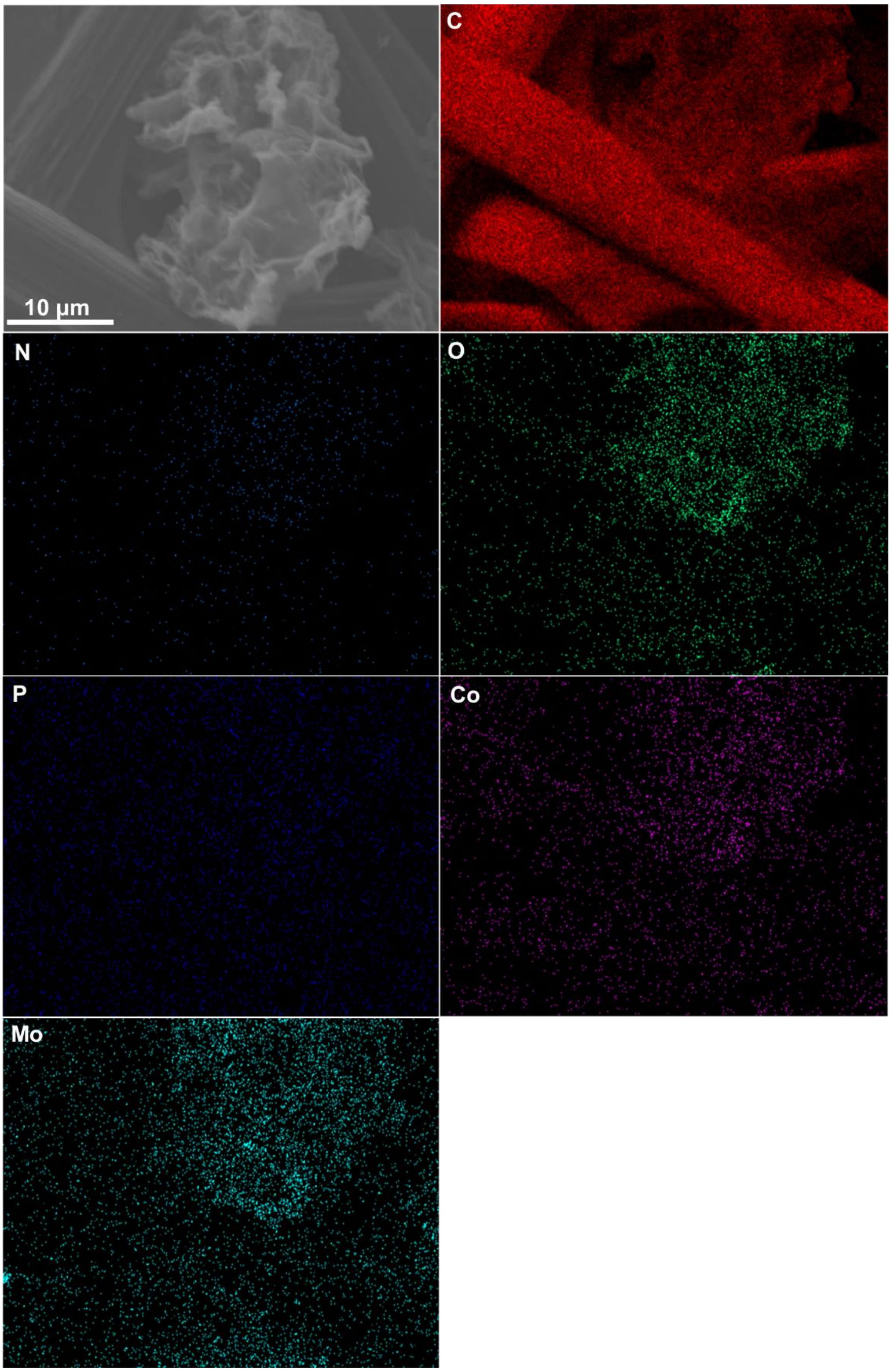

Figure S6. SEM image and corresponding EDS mapping of composite **4** modified carbon paper before water splitting, indicating the homogeneous dispersion of C, N, O, P, Co and Mo.

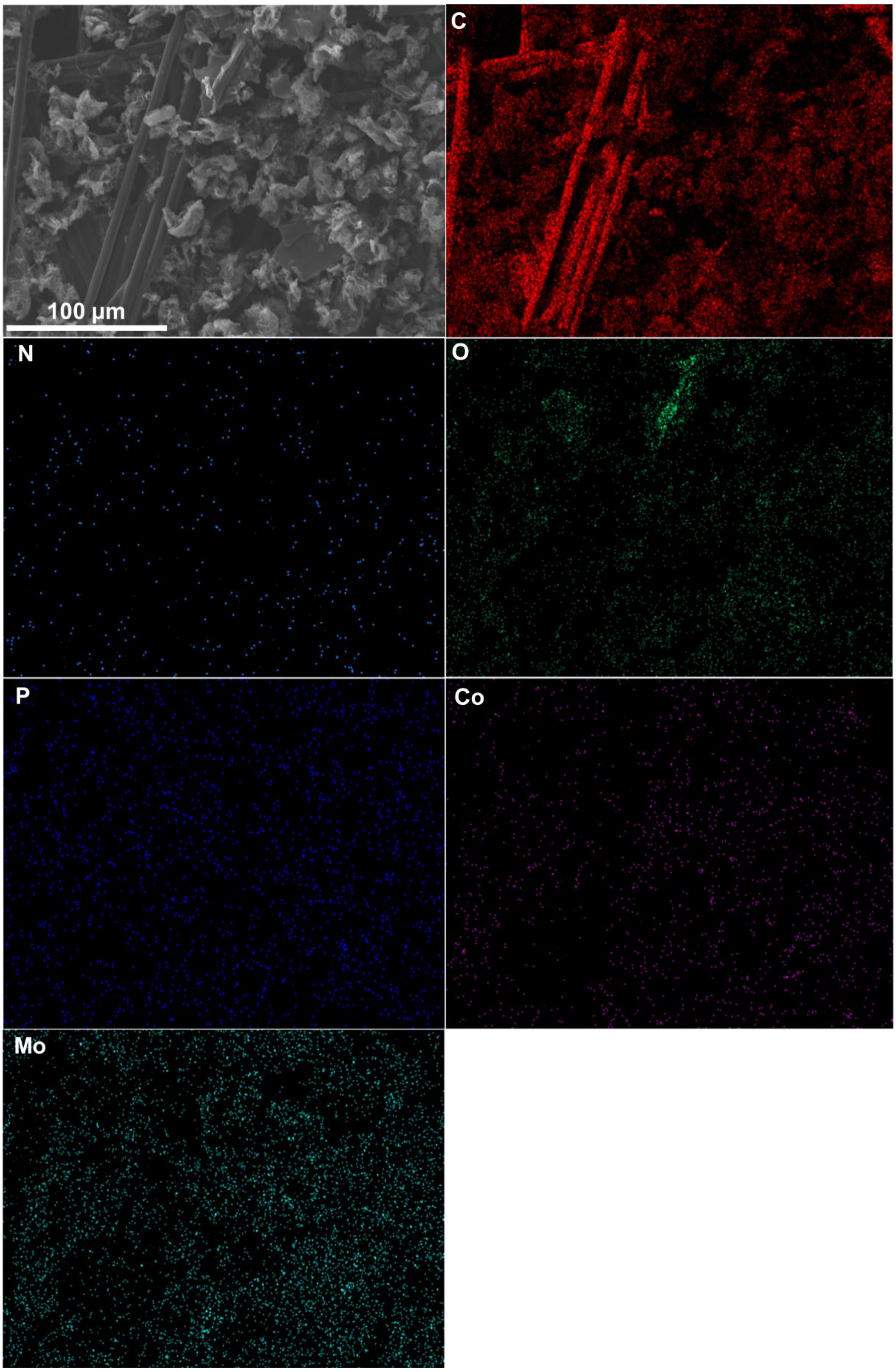

Figure S7. SEM image and corresponding EDS mapping of composite **4** modified carbon paper after water splitting on anode (OER), indicating the homogeneous dispersion of C, N, O, P, Co and Mo.

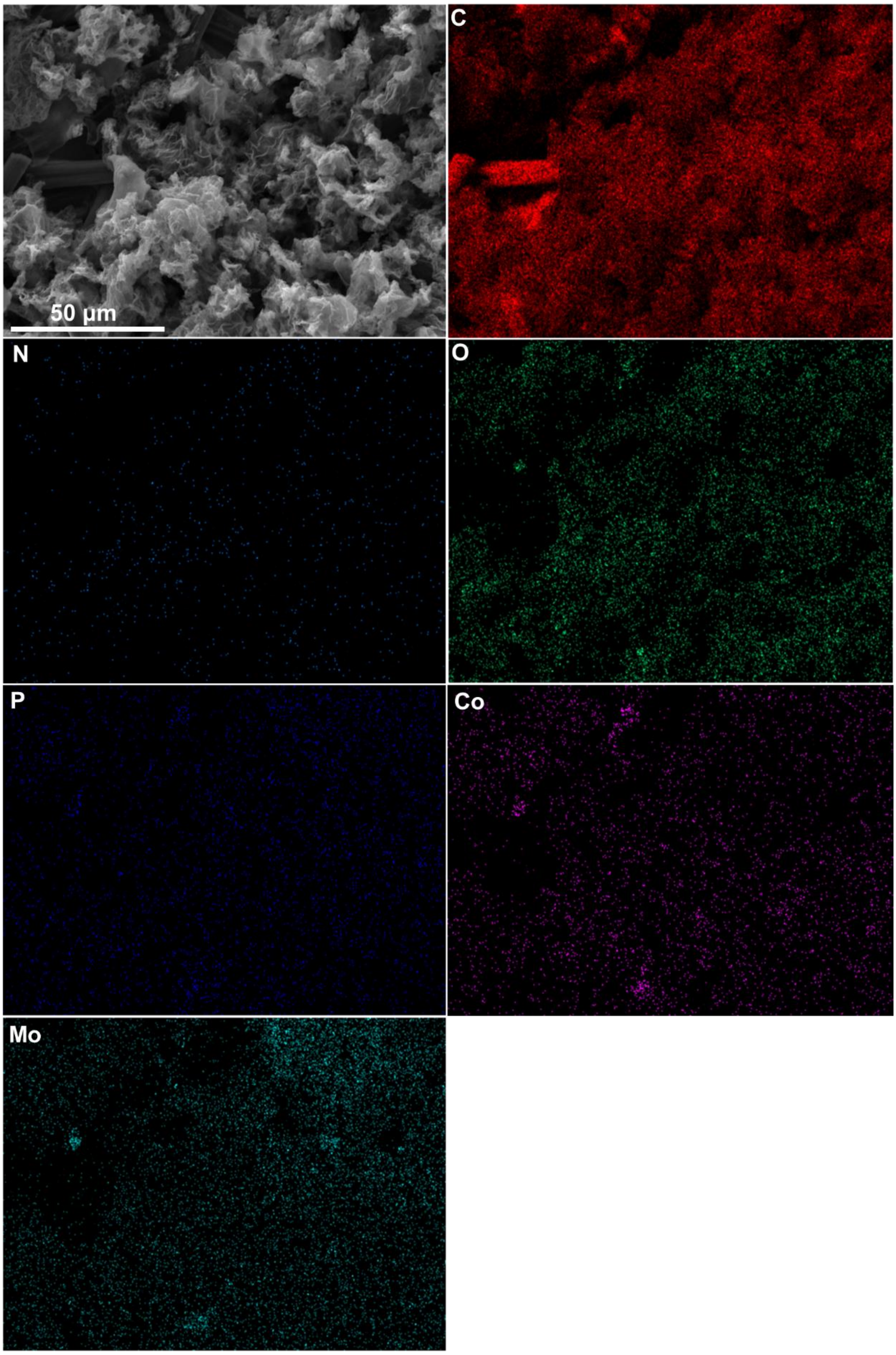

Figure S8. SEM image and corresponding EDS mapping of composite **4** modified carbon paper after water splitting on cathode (HER), indicating the homogeneous dispersion of C, N, O, P, Co and Mo.

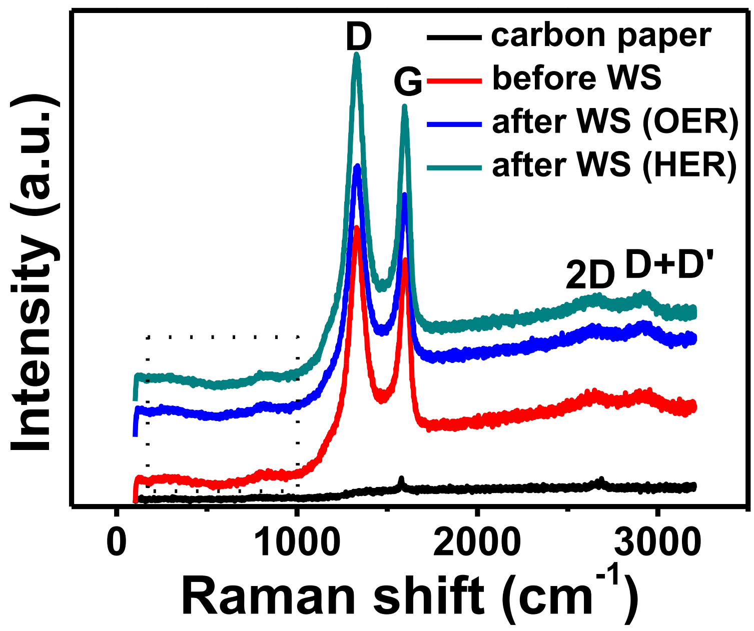

Figure S9. Raman spectra of composite **4-**modified carbon paper before and after water splitting (WS). The Raman spectrum of blank carbon paper was also added for comparison. It is shown that four peaks (D, G, 2D and D+D’ bands) assigned to graphene structures can be observed for **4** before and after WS. Besides, there are also some small peaks below 1000 cm^{-1} that may be due to the mixed carbide/oxide ($Co_6Mo_6C_2$/$Co_2Mo_3O_8$). The comparison shows that the composite **4** is structurally stable during WS.

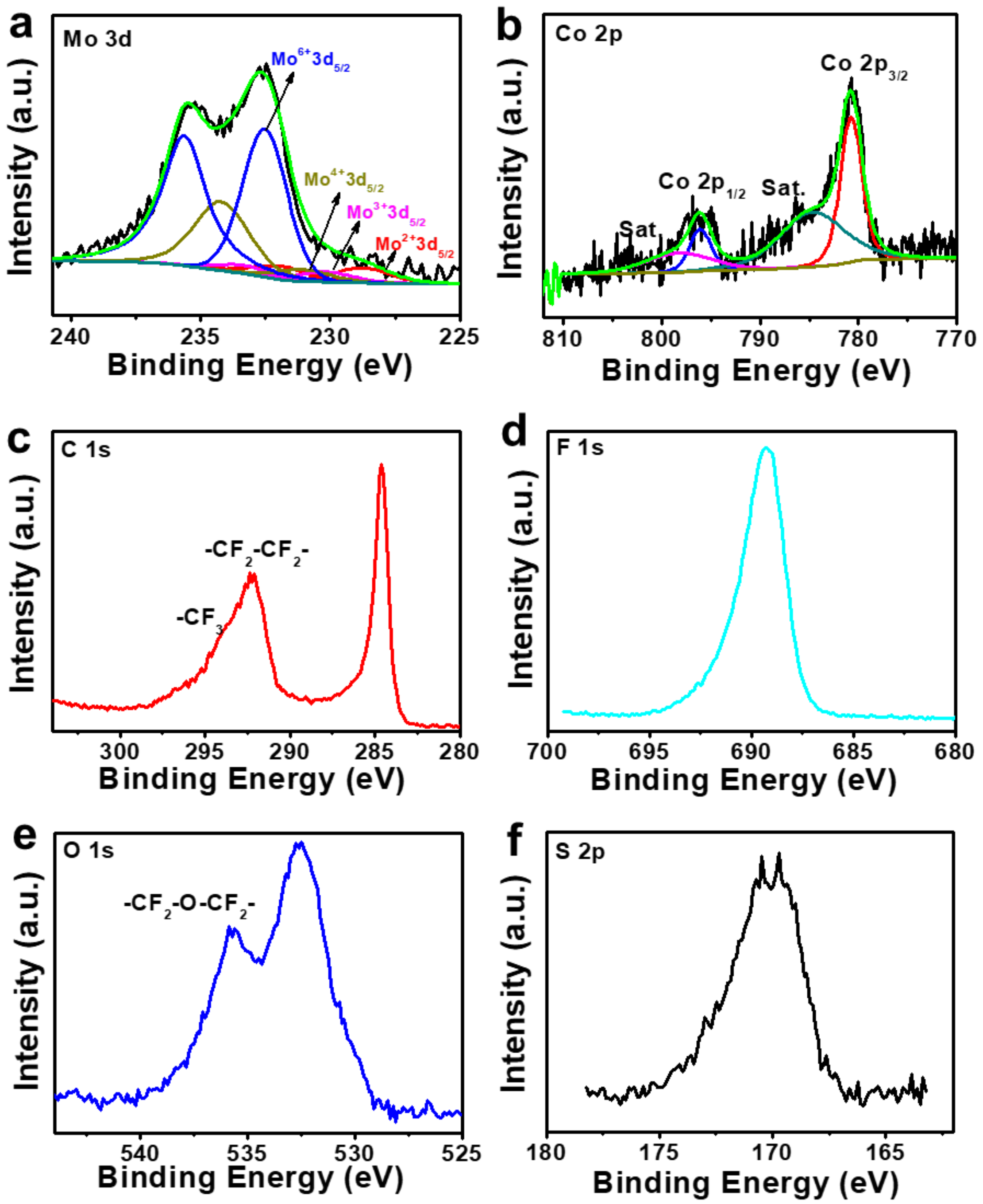

Figure S10. XPS spectra of composite **4-**modified carbon paper after water splitting (WS) in the anode (after OER). (a) Mo 3d, (b) Co 2p, (c) C 1s, (d) F 1s, (e) O 1s and (f) S 2p. The Mo 3d shows that the content of oxidation states of +2 and +3 were decreased that may be due to the electrochemical oxidation or surface oxidation of bimetallic carbides during electrocatalysis. The Co 2p still shows the dominant Co^{2+} species. It should be noted that the S/N ratio of P and N were low since the loading amount of the catalyst on the carbon paper was not high. The F and S were from Nafion used for preparing the modified electrode. The new peaks presented in the C 1s and O 1s were also from Nafion.[1]

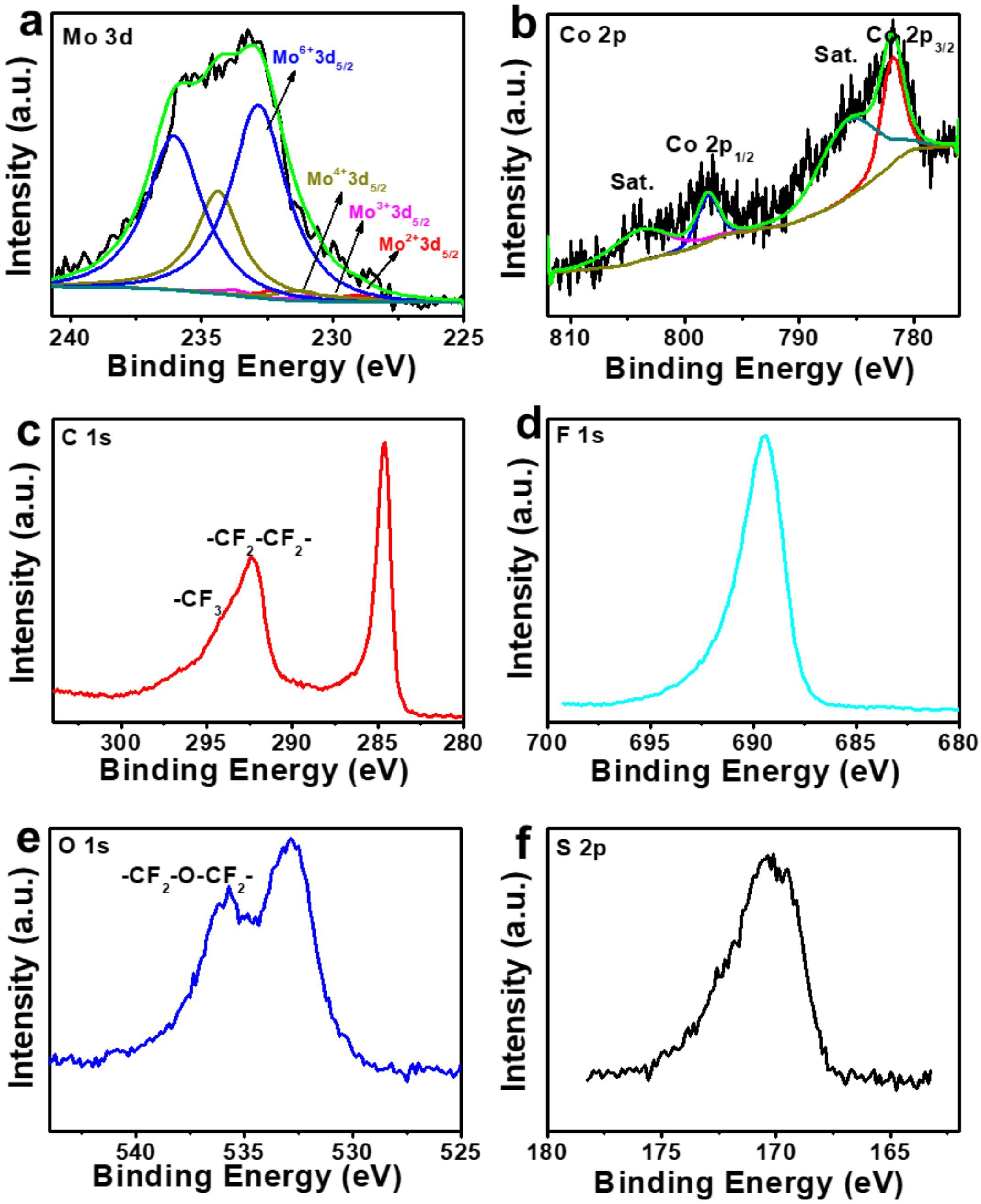

Figure S11. XPS spectra of composite **4**-modified carbon paper after water splitting (WS) in the cathode (after HER). (a) Mo 3d, (b) Co 2p, (c) C 1s, (d) F 1s, (e) O 1s and (f) S 2p. The Mo 3d shows that the content of oxidation states of +2 and +3 were decreased that may be mainly due to the surface oxidation of bimetallic carbides during electrocatalysis. The Co 2p still shows the dominant Co^{2+} species. It should be noted that the S/N ratio of P and N were bad since the loading amount of the catalyst on the carbon paper was not high. The F and S were from Nafion used for preparing the modified electrode. The new peaks presented in the C 1s and O 1s were also from Nafion.

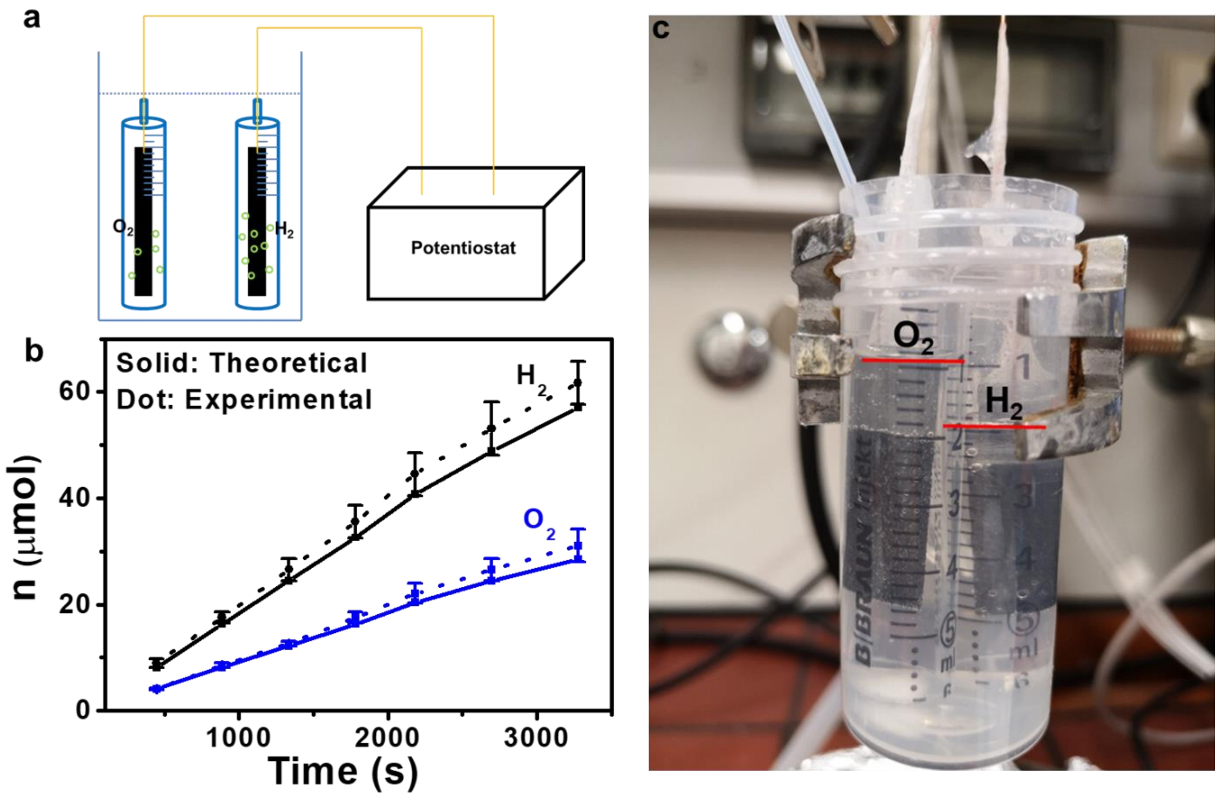

Figure S12. Volumetric quantification of the evolved H_2 and O_2. (a) Schematic drawing of the custom-made water electrolyzer, (b) Faradaic efficiencies of both H_2 and O_2, (c) Photograph of the custom-made water electrolyzer, showing the generated H_2 volume was twofold larger than that of O_2.

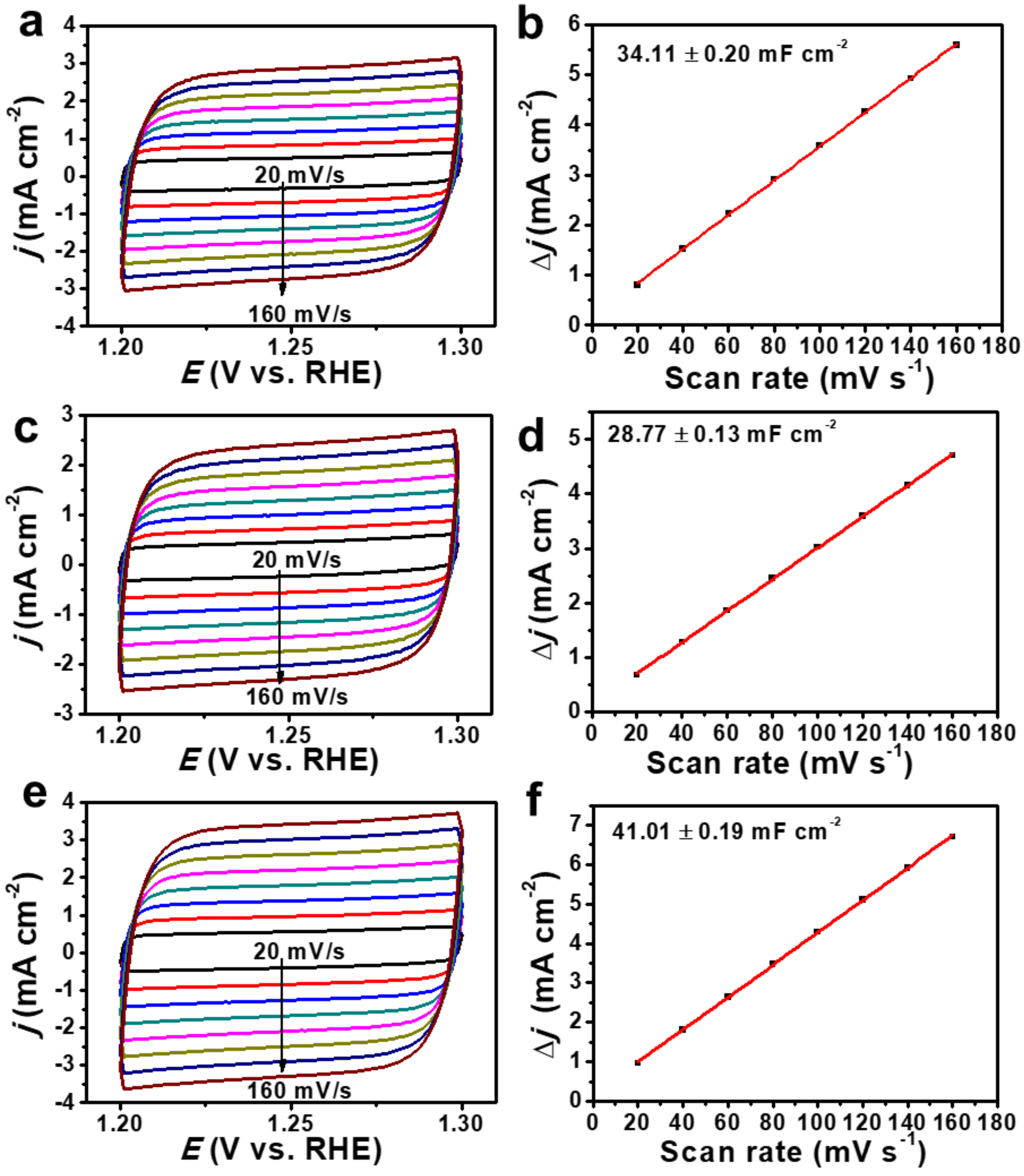

Figure S13. ECSA study of the catalysts. a,c,e CV curves for **2**, **3** and **4** at various scan rates. b,d,f The corresponding linear fitting plots of differences in current density ($\Delta j = j_a - j_c$) at a potential of 1.25 V depending on the scan rate. j_a: anodic current density; j_c: cathodic current density.

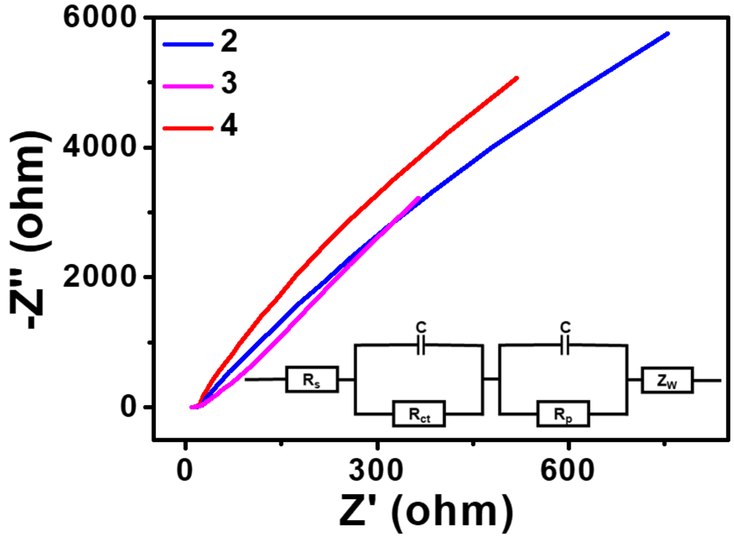

Figure S14. EIS test of composites **2**, **3** and **4**.

References

[1] A. K. Friedman, W. Shi, Y. Losovyj, A. R. Siedle, L. A. Baker, *J. Electrochem. Soc.* **2018**, *165*, H733–H741.
